# Dr. Sarah R. A. Dolley

**Published:** 1910-03

**Authors:** 


### OBITUARY

Dr. Sarah R. A. Dolley, of Rochester, died at her home in that city December 27, 1910, aged 80 years. Dr. Dolley was reputed to be the second woman to graduate from an American Medical College, having taken her medical degree in 1851 at a Syracuse Eclectic Medical College now extinct. She taught obstetrics in 1872-3 at the Woman's Medical College in Philadelphia, and served as a delegate to the American Medical Association at the Newport meeting, 1889.

